# Surface modification of Fe_2_O_3_ and MgO nanoparticles with agrowastes for the treatment of chlorosis in *Glycine max*

**DOI:** 10.1186/s40580-018-0155-0

**Published:** 2018-08-20

**Authors:** Abdul Azeez Nazeer, Sreelakshmi Udhayakumar, Saranpriya Mani, Mothilal Dhanapal, Sudarshana Deepa Vijaykumar

**Affiliations:** Nano-Bio Translational Research Laboratory, Department of Biotechnology, Bannari Amman Institute of Technology, Sathyamangalam, Erode, Tamil Nadu 638401 India

**Keywords:** Fe_2_O_3_ nanoparticles, MgO nanoparticles, Surface modification, Chlorosis, Chlorophyll, Biocompatibility

## Abstract

Surface modification of nanoparticles for biological applications is receiving enormous interest among the research community due to the ability to alchemy the toxic nanoparticles into biocompatible compounds. In this study, the agrowastes of *Moringa oleifera* and *Coriandrum sativum* were used to surface modify the magnesium oxide nanoparticles and ferric oxide nanoparticles respectively. The agrowaste amended magnesium oxide nano particles (AMNP) and agrowaste amended ferric oxide nanoparticles (AFNP) were characterized using scanning electron microscope, X-ray diffractometer, Fourier transformed-infra red spectroscope to justify the formation and surface modification of nanoparticles with the organic functional groups from the agro wastes. The surface modified nano particles were tested for their biocompatibility and ability to treat the chlorosis in *Glycine max*. On comparison between the two metal based nanoparticles, AMNP exhibited better chlorosis treating ability than the AFNP. Both the nano particles showed increased potency at minimal amount, 30 μg and the higher concentrations till 125 μg exhibited down run of the potency which was again enhanced from 250 μg of nanoparticle treatment to plants. Further the surface modified nanoparticles were assessed for biocompatibility on human embryonic kidney (HEK-293) cell line which proved that the cell lines are non-toxic to normal human cells. The size of the particles and the concentration is suggested to be responsible for the effective chlorosis treatment and the organic functional groups responsible for the reduction of toxicity of the particles to the plants.

## Introduction

Iron oxide nanoparticles (Fe_2_O_3_) are one of the most widely studied metallic nanoparticles, which have a broad spectrum of applications in controlled drug release, medical diagnostics, separation technologies and environmental engineering due to their novel properties such as enhanced surface-to-volume ratio, activated surface area and inherent biocompatibility [1, 2]. However, there are phyto-toxicity issues reported on the chemically synthesized iron oxide nano particles for inhibiting the growth of some algal species [3]. In a concentration depended toxicity study by Liu et al. [4] on maghemite nanoparticles, 20–70 ppm of maghemite nano particles suppressed the growth of lettuce, while at 1 ppm the particles enhanced the growth of the plants. Most of the dicots and monocots uptake iron from the soil using a system termed ‘strategy I’ [5]. The strategy I plants respond to iron-limiting stress in three steps. (A) releasing proton and organic acids to acidify the rhizosphere, driving more Fe(III) into solution, (B) reducing Fe(III) to Fe(II) at the root surface via Fe(III)-chelate reductase, and (C) transporting Fe(II) across the root epidermal cell membrane via activation of a high-affinity Fe(II)-transport system [6]. The Fe(III) is insoluble in neutral, alkaline, and calcareous soils making them unavailable for plants to be taken in leading to iron deficient chlorosis [7]. Hence the preparation of Fe(III) nano particles at a size of less than 30 nm and amending their surface with organic functional groups from the agrowastes can be used to treat the chlorosis, the iron deficiency in plants.

Chlorosis is a common widespread plant disease caused due to the lack of chlorophyll in plants. This lack of chlorophyll is reported to be influenced by poor iron uptake by plants from the soil. Treatment of chlorosis is being focused only on the perspective of deficiency of iron and extensive works are conducted on treating plants with iron [8–12]. But the core of the chlorophyll that is responsible for the photosynthesis has magnesium in it. Lack of chlorophyll is the major symptom of chlorosis. Hence an attempt is made to treat the chlorosis infected plants with surface amended biocompatible iron oxide and magnesium oxide nano particles independently and compare the efficacy of the treatment.

Surface modification of iron nanoparticle with the organic functional groups is being studied extensively for various applications [13–17]. There are very few reports on the biologically surface modified synthesis of magnesium nano particles [18]. There have been various surface modification approaches such as citrate gel method for thermosensitive polymer coating [19], surfactant template method for energy storage [20], sol–gel approach for ceramic coating [21]. In this exploration, we have used the agrowastes of *Coriandrum sativum* for the synthesis of Fe_2_O_3_ nanoparticles and *Moringa oleifera* for the synthesis of MgO nanoparticles. The functional groups in the agro waste extracts reduce the metallic salt solution to precipitate the metallic oxide nanoparticles. These metallic nanoparticles have their surface amended with organic functional groups like amide, aldehydes, ketones that nullify the invasiveness and toxicity of the nanoparticles. The agrowaste amended ferric oxide nano particles (AFNP) and agrowaste amended magnesium oxide nano particles (AMNP) were then tested on the plants for their ability to treat chlorosis.

The results were fruitful in treating the chlorosis and this approach can be employed and explored further for a better green sustainability.

## Materials and methods

### Materials

Agro wastes of *M. oleifera* and *C. sativum* were collected from nearby hostel kitchen in Sathyamangalam, Erode. Collected plant parts were washed and Soxhlet extracted at 90 °C with distilled water and the aqueous extracts obtained were stored at 20 °C and used when required. Ferric chloride, magnesium chloride, sodium hydroxide were purchased from Sigma Aldrich (USA) local suppliers and distributors. *Glycine max* seeds were purchased from local markets in Sathyamangalam, Erode, India. Human embryonic kidney cell line HEK-293 (ATCC CCL1573) was obtained from National Centre for Cell Science, Pune.

### Synthesis of AFNP and AMNP

The synthesis of AFNP and AMNP was carried out by adding 0.1 M NaOH solution and plant extracts, *C. sativum* and *M. oleifera* extract drop wise to the stirring solutions of 0.1 M FeCl_3_·7H_2_O and MgCl_2_ in 0.1 M NaOH solutions respectively. The addition was titrated until the formation of AFNP and AMNP. Formation of AFNP was observed by colour change from brown to reddish black while formation of AMNP was observed by colour change from white to pale green. Solutions were centrifuged at 11,180*g* for 10 min. Supernatants were discarded and the pellets obtained were air dried.

### Soil analysis for pH

Soil was collected from the farmlands in Sathyamangalam where chlorosis is prevalent. Soil sample dispersed in water was tested with pH meter and found to be 8.

### Field study with *Glycine max*

Field study was conducted on *Glycine max* that is highly susceptible to chlorosis. Seeds were germinated in batches and the soil was added with AFNP and AMNP at varying concentration (500 µg, 250 µg, 125 µg, 62.5 µg, 31.25 µg and 15.625 µg)/100 mL of watering individually. A control was grown without subjecting to nanoparticle treatment and all the plants were planted in an herbal garden for further growth.

### Chlorophyll content of *Glycine max* leaves

The fresh leaves of nano particles treated and untreated *Glycine max* plants were collected and solvent extracted with 5 mL 80% acetone solution using prechilled pestle and mortar. The mixture was centrifuged at 5590*g* at 4 °C for 10 min, and the supernatant was collected. Absorbance of the supernatant was measured at 450 nm using UV–visible spectrometry. Supernatants from untreated plant leaves were taken as control. The experiment was performed in triplicates.

### Characterization of AFNP and AMNP

#### Fourier transform infra-red spectroscopy analysis

The FTIR spectra were recorded on Perkin Elmer Spectrum RXI FTIR Spectrometer at room temperature. Briefly, the synthesized nanoparticles were coated onto KBr crystal wafers and dried before measurements. The IR spectra were noted in the wavelength region of 4000–400 cm^−1^ and assigned peak numbers. Background correction was made using a reference blank KBr pellet.

#### X-ray diffraction analysis

The crystal conformation and size of AFNP and AMNP were studied using Bruker AXS D8 Advance X-Ray Diffractometer, with a 1.5406 Å wavelength Cu-Kα beam operated at 40 kV and 35 mA.

#### Scanning electron microscopy

The surface morphologies of AFNP and AMNP were studied using JSM-6390 (VEGA3 TESCAN LMU-USA) Scanning electron microscope, with an accelerating voltage of 20 kV. The samples for SEM observations were prepared by mounting small amount of AFNP and AMNP independently onto the surfaces of clean carbon chips and analyzed with incident electron beam.

### Cytotoxic studies

MTT Assay was performed as per the protocol by Mosmann 1983 [22]. Briefly, human embryonic kidney HEK-293 (ATCC CCL1573) cells were incubated and maintained as monolayers in 25 sq. cm flasks using Iscove’s modified Dulbecco’s medium with 10% fetal bovine serum. One hundred microlitres per well of HEK-293 cell suspension were seeded into 96-well plates at plating density of 10,000 cells/well and incubated. After 24 h, the cells were treated with serial concentrations of AFNP and AMNP independently. After 48 h of incubation, 15 µl of MTT 3-(4,5-dimethylthiazol-2-yl)-2,5-diphenyltetrazolium bromide (5 mg/ml) in phosphate buffered saline (PBS) was added to each well and incubated at 37 °C for 4 h. The medium with MTT was then flicked off and the formed formazan crystals were solubilized in 100 µl of DMSO and then the absorbance was measured at 570 nm. The percentage cell growth was then calculated with respect to control as follows

$$\% {\text{ cell growth }} = \, \left[ {\text{A}} \right]{\text{test}}/ \left[ {\text{A}} \right]{\text{control }} \times \, 100$$

[A] = absorbance at 570 nm.

## Results

### Synthesis of AFNP and AMNP

Polyphenols present in the plant extracts can reduce the metallic salts into metallic or metal oxide nanoparticles [23]. *Coriandrum sativum* and *M. oleifera* have polyphenolic groups that can reduce the metallic chloride salts into metallic oxides and precipitate as nano particles with the increase of pH by NaOH. Phenolic groups on donating the OH^−^ to the solution catalyze the reduction of metallic salts into metallic hydroxides which on further condensation forms the metallic oxide nanoparticles [24, 25]. The color change from crimson red to blackish red precipitate marked the formation of ferric nano particles while the pale green precipitate marked the formation of magnesium oxide nanoparticles, both surface amended with plant extracts respectively.

### FTIR analysis

#### FTIR analysis of AFNP

**Fig. 1:**
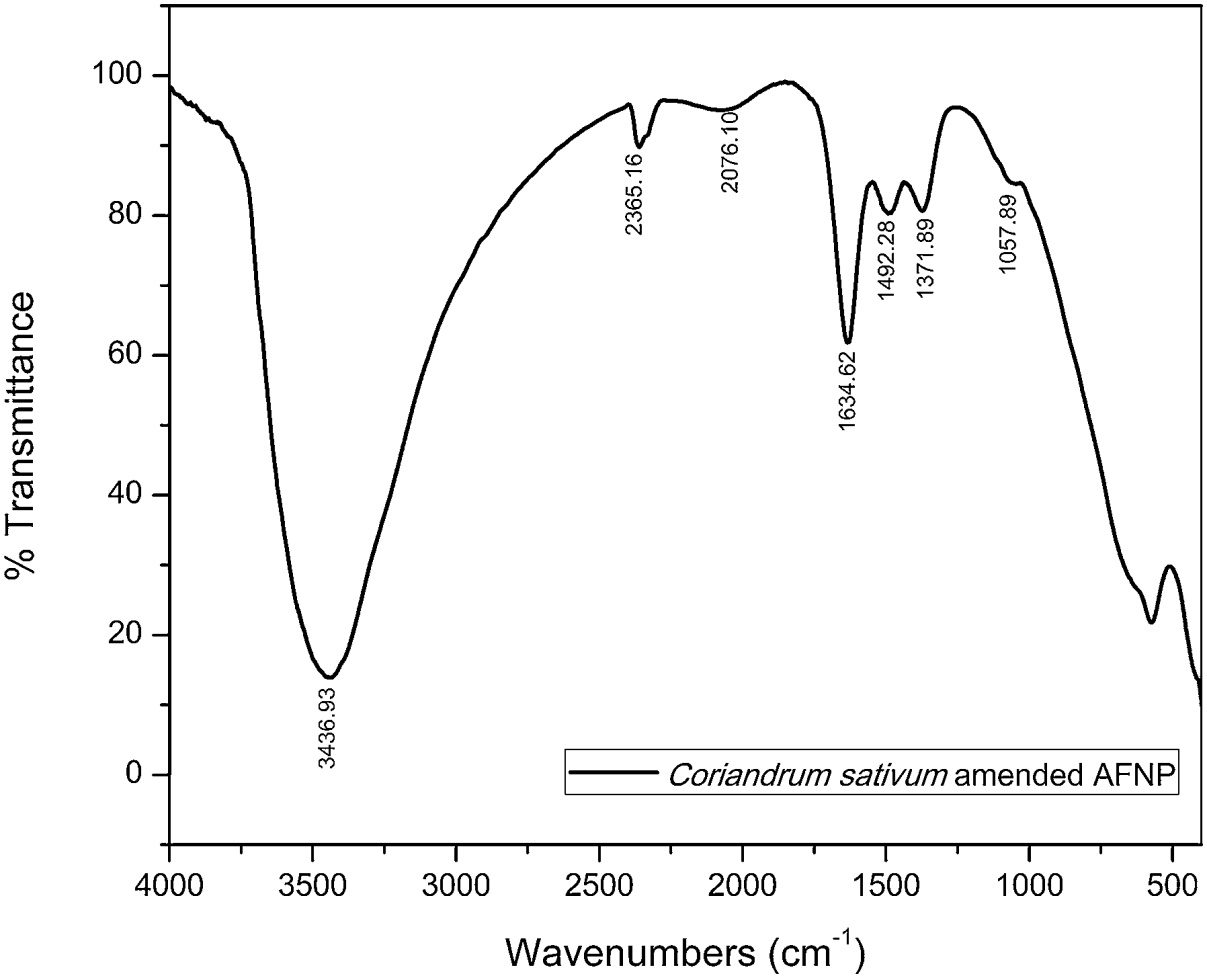
FTIR spectrum of AFNP

The vibrational frequencies were studied using FT-IR to determine the surface functional groups of the AFNP. From the peaks (Fig. 1), the presence of hydroxyl and carbonyl bonds is evident justifying the presence of organic functional groups from the *C. sativum* agro waste on the surface of ferrous oxide nanoparticle. The broad peak at wave number 3437 cm^−1^ corresponds to the presence of exchangeable protons from strong primary amide N–H stretching bond. The peak at 1635 cm^−1^ corresponds to the C=O vibrations from terpenoids [26]. From an analogous work by Mariselvam et al. [27] absorption band at 1638 cm^−1^ was claimed to be responsible for the formation of AgNPs by the terpenoids in the *Cocos nucifera* extract. In a similar fashion, the probability of amendment on the surface of ferric oxide nano particles could be due to the terpenoids present in the *C. sativum* extract. Wavenumbers 1372 cm^−1^ corresponds to Sp3 C–H bend and 1058 to Sp3 C–N stretch. The other organic functional groups on the surface reduce the invasiveness of the nanoparticle thence making it non-toxic to plants.

#### FTIR analysis of AMNP

**Fig. 2:**
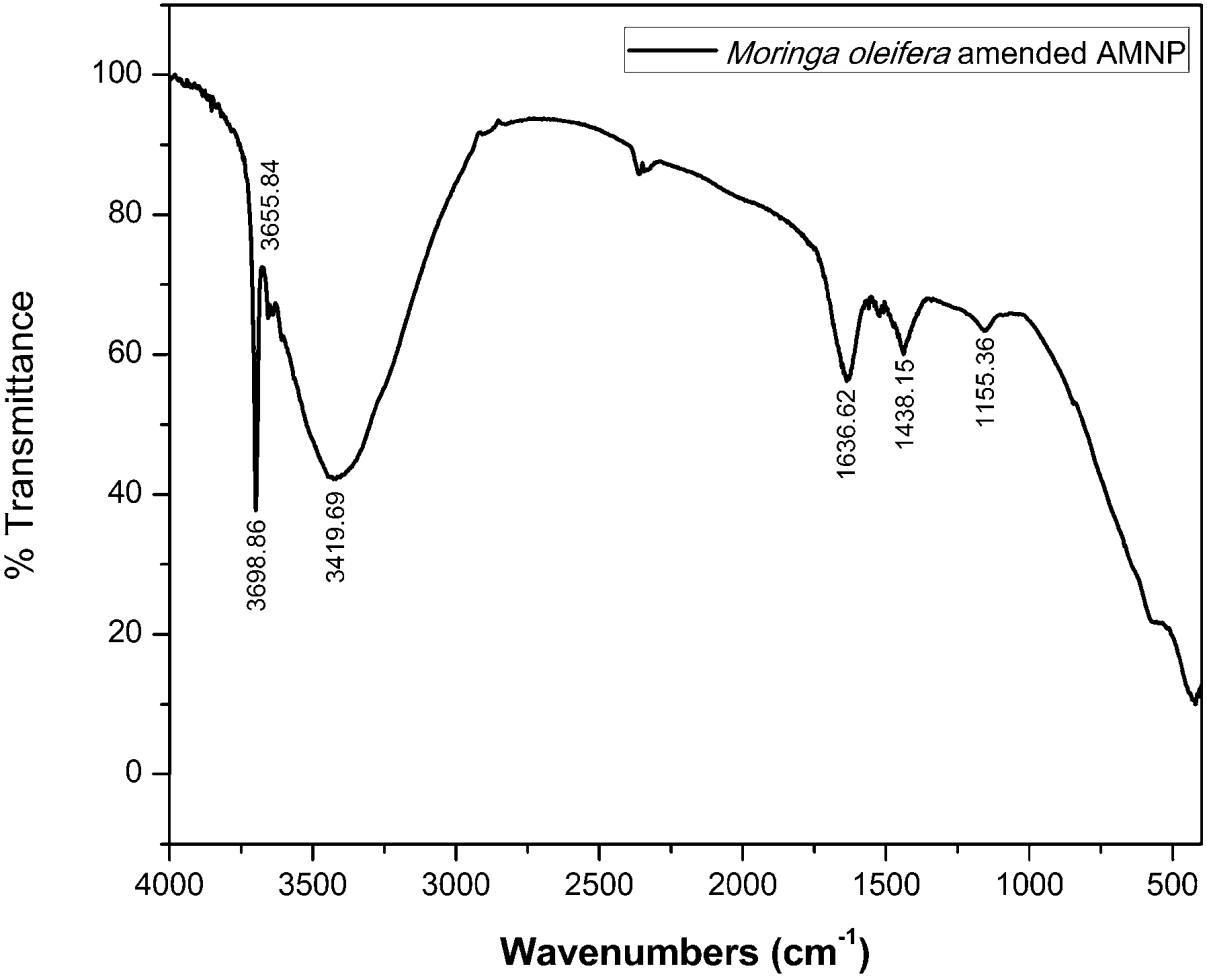
FTIR spectrum of AMNP

The vibrational frequencies were studied using FTIR to find the functional groups on the surface of the AMNP. From the peaks (Fig. 2), the presence of hydroxyl and carbonyl bonds is evident which provides the possibility of binding of organic functional groups from the *M. oleifera* agro waste on magnesium oxide nanoparticle. Wave numbers 3420 cm^−1^ corresponds to strong –OH and primary amide (=NH) group from protein, fatty acids, carbohydrates and the lignin units [28]. Since the agro waste used here was the stem part which was rich in fibers lignin, the stretch vibration can be attributed by the –OH of the lignin. The vibrational frequency at 1637 cm^−1^ corresponds to Sp2 C=N (double bond stretch carbonyl) groups such as esters, ketones and aldehydes [29]. The wave numbers 1560 cm^−1^, 1522 cm^−1^ corresponds to the asymmetric nitro stretch and its presence may be due to the stretching of the C–N linkage and the deformation of the N–H linking proteins [28].

### XRD analysis

**Fig. 3:**
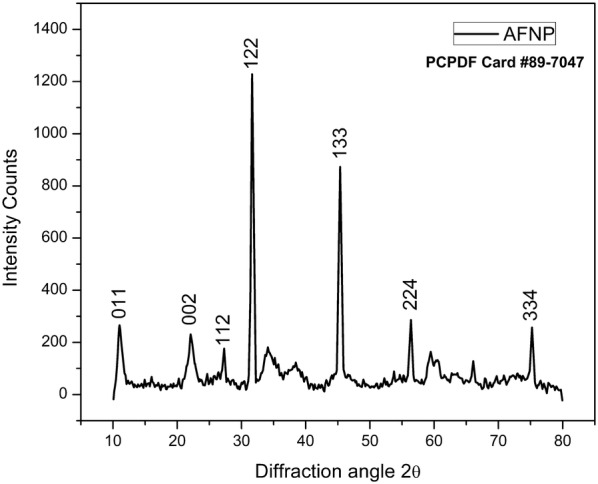
XRD pattern of the AFNP

**Fig. 4:**
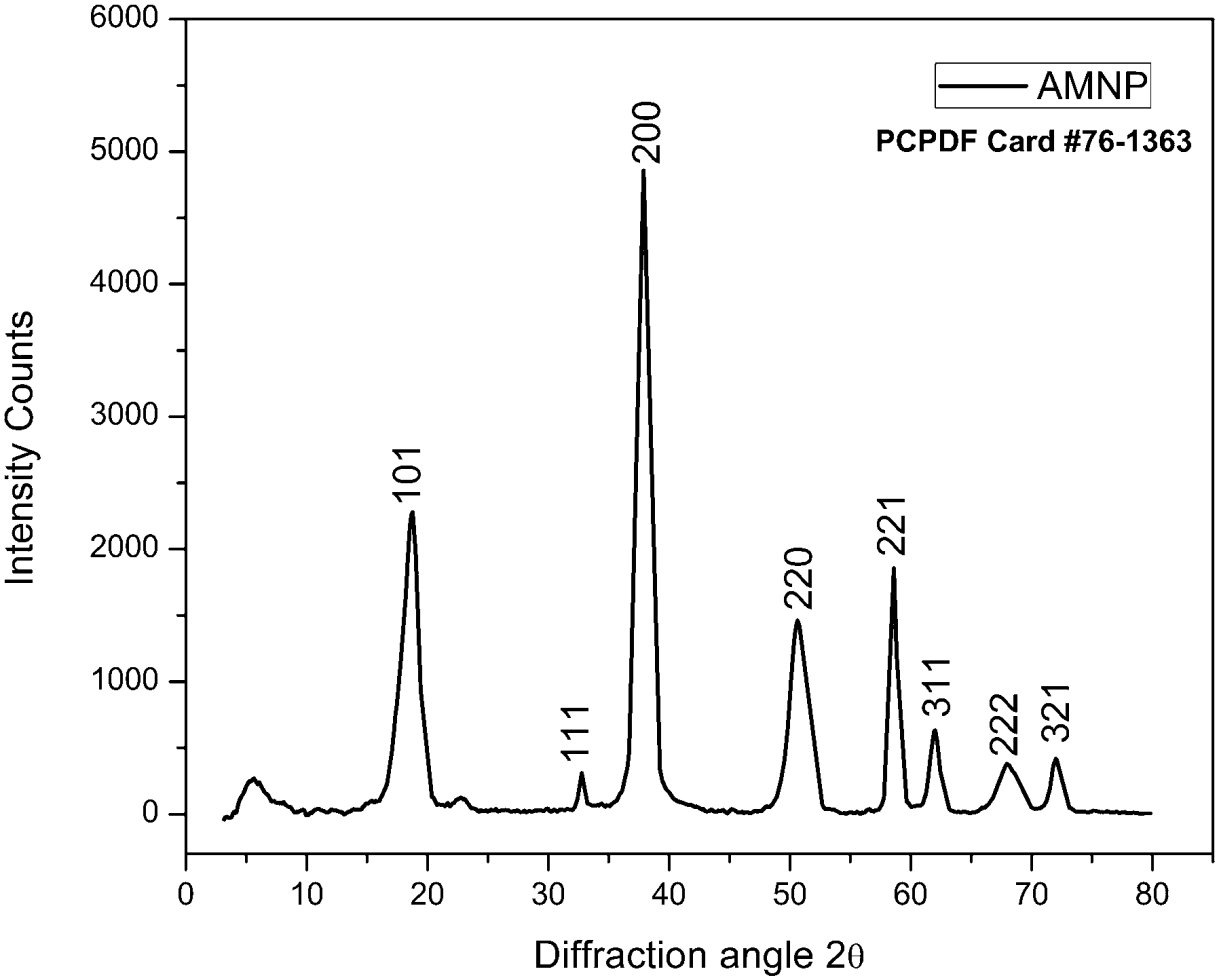
XRD pattern of the AMNP

The major X-ray refraction crystallographic planes corresponding to each 2θ values are indexed by 011(11.03), 002(22.139), 112(27.326), 122(31.674) and 133(45.37) for AFNP in Fig. 3. The results are in agreement with the XRD standard for the ferric oxide nanoparticles in PCPDF win Card No. 89-7047. The crystal lattice is BCC, orthorhombic as per the detailed explanation by Tronc et al. [30]. For the AMNP, the major x-ray reflection crystallographic planes corresponding to each 2θ values are indexed by 101(18.726), 111(32.11), 200(37.89), 220(50.64) 221(58.59), 311(61.45), 222(68.24) and 321(72.25) in Fig. 4. The results are in agreement with the XRD standard for the magnesium oxide nanoparticles in PCPDF win Card No. 76-1363. The crystal lattice of AMNP is BCC, cubic as per the deliberation by Vannerber [31]. This AMNP peak pattern is similar to the peak pattern of Wang et al. [32].

Crystal size of the synthesized nanoparticles was calculated from the Debye–Scherrer equation

$${\text{D }} = {\text{ K}}\lambda / \, \beta { \cos }\theta$$

where D = crystal size, β = full width half maximum of the peak, λ = X-ray wavelength (1.54 Å), K = shape factor which is always close to unity (0.94).

Crystal size of the AFNP calculated from the Debye–Scherrer equation is found to be 11.26 nm and that of AMNP to be 7.54 nm.

### SEM analysis

**Fig. 5:**
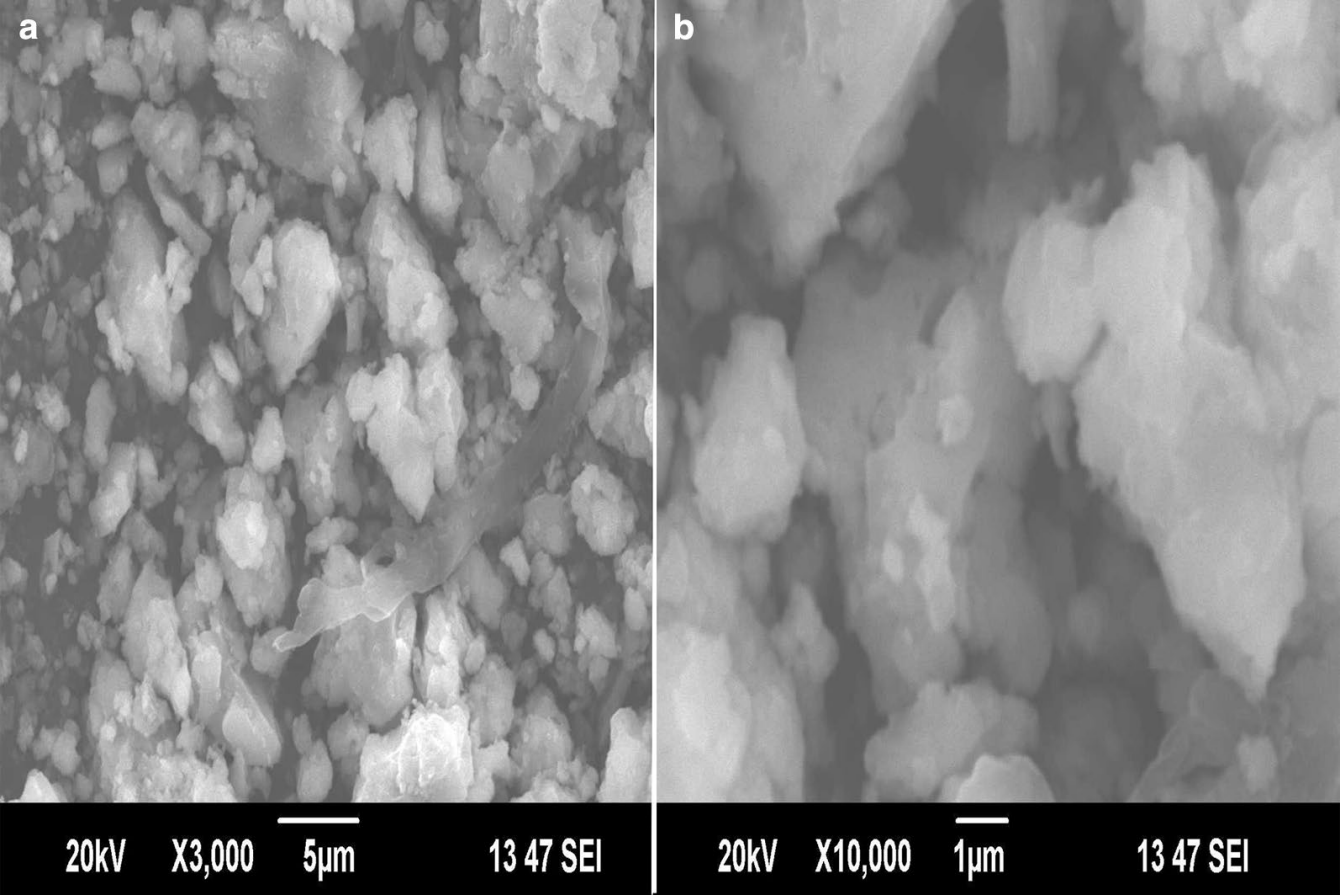
SEM micrograph of AFNP. **a** The magnification scale of 5 μm shows the orthorhombic crystals, **b** the magnification scale of 1μm shows the size of the crystals that are in nanometric scale

**Fig. 6:**
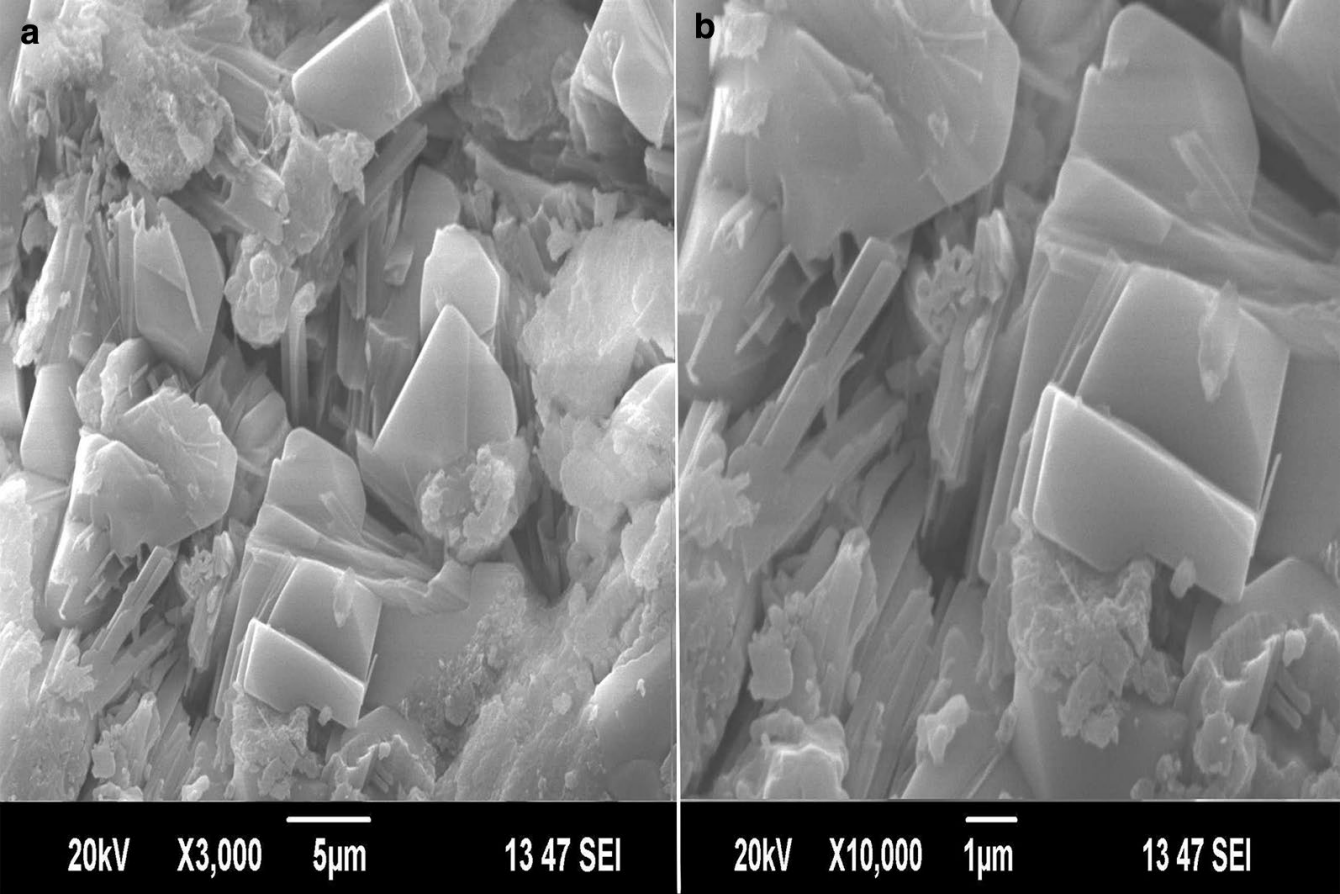
SEM micrograph of AMNP. **a** The magnification scale of 5 μm shows the cubic crystals, **b** the magnification scale of 1μm shows the size of the crystals that are in nanometric scale

The scanning electron microscopic images (Fig. 5a, b) of AFNP show orthorhombic crystals in nanometer scale. Sayed and Polshettiwar [33] studied the different shapes and their corresponding properties of the iron oxide nanoparticles. They have reported that the property of the hexagonal and orthorhombic iron nanocubes are slightly thermosensitive that on heating would deform to form microcubes [33]. This morphology result is also analogous to previous biologically synthesized iron oxide nanoparticle reports [34–36]. Scanning electron microscopic images (Fig. 6a, b) of AMNP show the nanocubes of synthesized magnesium oxide nanoparticles cubic. They also exhibit more colloidal property than the AFNP, forming flakes. These flakes were dispersed in water on manual stirring. Similar morphology was observed by Wang et al. [32]. In an earlier work by Demirci et al. [37] spherical MgO nanoparticles were observed when synthesized by Flame Spray Pyrolysis (FSP) method. Though the spherical nanoparticles are better for being conducted by plant organs, the preparatory method makes them toxic to the plant tissues.

### Chlorophyll content test

In the chlorophyll content test, there was an appreciable increase in chlorophyll at the minimal volume of 30 μg of both AFNP and AMNP individually. But the chlorophyll content reduced with increase in AFNP and AMNP amount from 60 to 125 μg. This case was also found in the previous research work by Wang et al. [38] where there was increase at minimal concentration and decrease in the chlorophyll content with increase in iron oxide nano particles. In their study, they chemically synthesized nano ferric oxide and tested the same to improve the chlorophyll content in watermelon. They have hypothesized that the decrease in chlorophyll content might be due to aquatic environment that restrains the uptake of iron. In this research, the reduction in chlorophyll content at higher concentrations can be due to accumulation of nano particles at certain concentrations thence making only partial nano particles for transport from root to leaves. This accumulation hypothesis can be substantiated by Bombin et al. and Ma et al. [39, 40]. They testified that plant roots take up the iron oxide from the soil and distribute them throughout the plant tissues but the accumulation of nanoparticles in roots is more than in leaves. They have claimed that this accumulation was due to the size of the particles were large. It is to be noted in this study that the chlorophyll content was increased in concentration 250 μg and maximum at 500 μg. The dicotyledon plant probably followed strategy I to uptake the AFNP by reducing the Fe(III) complex using the reductase enzyme. But the production of Fe(III)-chelate reductase enzyme is not dependent on the concentration of the nanoparticles, since the magnesium nano particles also exhibited the same pattern of treatment.

**Fig. 7:**
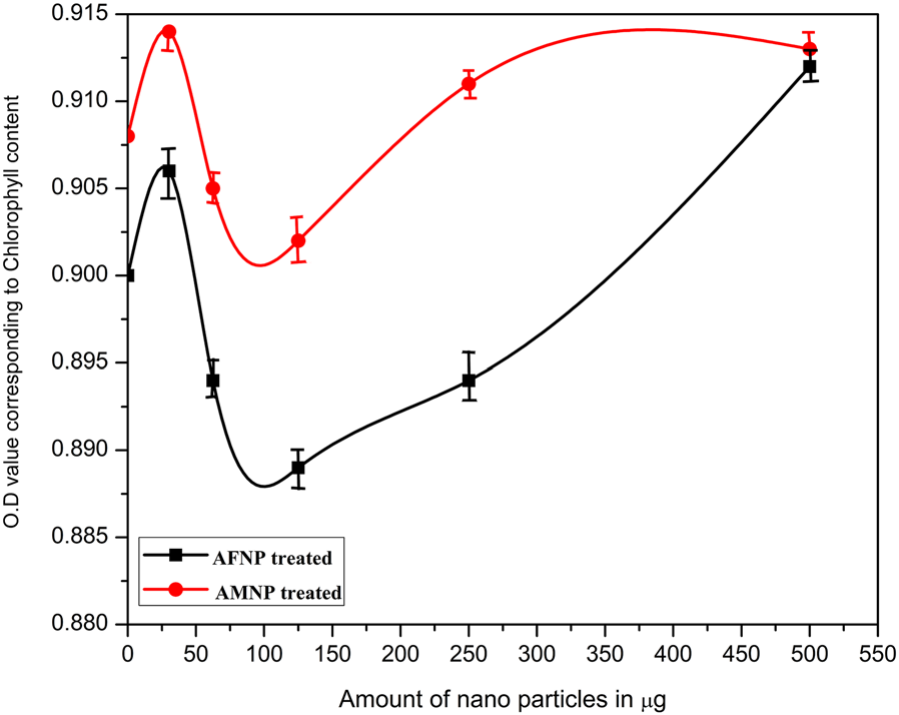
Chlorophyll content curve with respect to the amount of AFNP and AMNP

From Fig. 7, it is evident that treating plants with *M. olifera* amended Magnesium oxide nano particles is efficient than *C. sativum* amended hematite (Fe_2_O_3_) nano particles. Though the amendment varies between the two metallic oxide nanoparticles, it is to be noted that, *M. olifera* or *C. sativum* as such do not produce any anti-chlorosis activity. Hence the chlorophyll enhancement is attributed solely to the core metallic oxide nano particles while the amendment is only for the purpose of phyto compatibility and enhanced iron supplement.

### Cytotoxic studies

**Fig. 8:**
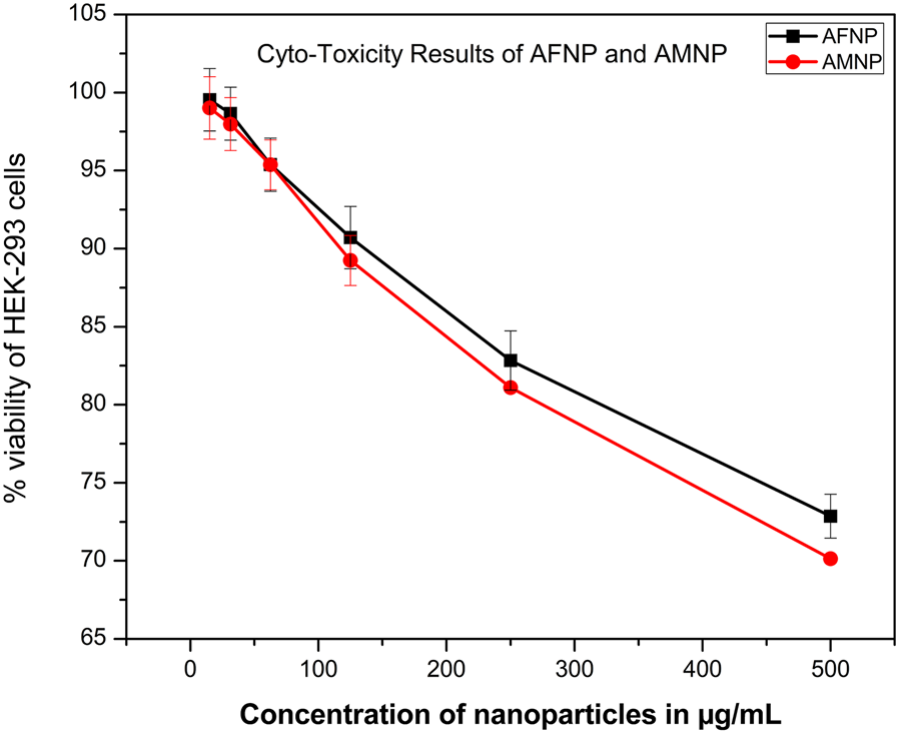
Graphical representation of the toxicity of the nanoparticles on HEK-293 cell line through MTT assay

The biocompatibility of the AFNP and AMNP was analyzed on human embryo kidney (HEK-293) cell line, by employing MTT assay, and responses are presented as the percent cell viabilities after treating with varying concentrations of AFNP and AMNP ranging from 15 to 500 μg/mL for 24 h. From Fig. 8, it is evident that the cells survive more than 50% even at the highest concentration of 500 μg/mL. The 90% viability concentration or 10% lethal concentration (LC_10_) of AFNP and AMNP was simulated to be at 209.47 μg/mL and 206.11 μg/mL respectively. The 50% lethal concentration (LC_50_) of AFNP and AMNP was never reached within the test range of the nanoparticles justifying that the particles are biocompatible. Similar result was observed on the same HEK-293 cell line by Kavya et al. where iron oxide nanoparticle was prepared using agrowastes of various plants [41]. The cytotoxicity of bacterial enzymatic synthesis ferric oxide nanoparticles was evaluated against the cancer cell lines and normal vero cell line and observed that the biologically synthesized iron oxide nanoparticles are non-toxic to normal cells but were potentially lethal to the cancer cells [42]. The cytotoxicity of the chemically synthesized MgO nanoparticles was observed by Mahmoud et al. [43] where the LC_50_ was attained at less than 300 μg/mL.

## Conclusion

Iron rich *M. olifera* agro waste extract was used to amend the surface of magnesium oxide nanoparticles and the magnesium containing *C. sativum* agro waste extract was used to amend the surface of the hematite (Fe_2_O_3_, iron oxide) nanoparticles. The surface modified nanoparticles were tested to treat the chlorosis in *Glycine max* plant by improving the chlorophyll content. On the evaluation, it was found that the nanoparticles were better absorbed by roots at minimal amount, 30 μg and we claim that to be the ideal concentration of like nano particles (of size 50–90 nm) to treat the plants without harming the environment. Also we conclude that the biological synthesis of nanoparticles would yield nontoxic biocompatible product and suggesting that the concentration of the nanoparticles or micronutrients is not responsible for the amount of production of Fe(III) reductase enzyme.

## Abbreviations

AMNP: agrowaste amended magnesium oxide nano particles
AFNP: agrowaste amended ferric oxide nano particles
FTIR: Fourier transform infra-red spectroscopy
XRD: X-ray diffraction
MTT: 3-(4,5-dimethylthiazol-2-yl)-2,5-diphenyltetrazolium bromide
HEK: human embryonic kidney cells

## References

[1] He S, Feng Y, Ren H, Zhang Y, Gu N, Lin X (2011). J. Soils Sediments.

[2] Wang Y, Hu J, Dai Z, Li J, Huang J (2016). Plant Physiol. Biochem..

[3] Demir V, Ates M, Arslan Z, Camas M, Celik F, Bogatu C, Can SS (2015). Bull. Environ. Contam. Toxicol..

[4] Liu R, Zhang H, Lal R (2016). Water Air Soil Pollut..

[5] Marschner H (1995). Mineral nutrition of higher plants.

[6] Yuan Y, Wu H, Wang N, Li J, Zhao W, Du J, Wang D, Ling HQ (2008). Cell Res..

[7] Prasad PVV, Djanaguiraman M (2017). Encyclopedia of applied plant sciences.

[8] Lopez-Rayo S, Hernandez D, Lucena JJ (2009). J Agric. Food Chem..

[9] Lucena JJ (2006). Iron nutrition in plants and rhizoshpheric micro-organisms.

[10] Martín-Fernández C, Solti Á, Czech V, Kovács K, Fodor F, Gárate A, Hernández-Apaolaza L, Lucena JJ (2017). Plant Physiol. Biochem..

[11] O’Rourke JA, Graham MA, Vodkin L, Gonzalez DO, Cianzio SR, Shoemaker RC (2007). Plant Physiol. Biochem. PPB.

[12] Vercelli M, Gaino W, Contartese V, Gallo L, Carlo SD, Tumiatti V, Larcher F, Scariot V (2015). Acta Scientiarum Polonorum Hortorum Cultus.

[13] Fazlzadeh M, Rahmani K, Zarei A, Abdoallahzadeh H, Nasiri F, Khosravi R (2017). Adv. Powder Technol..

[14] Irshad R, Tahir K, Li B, Ahmad A, Siddiqui RA, Nazir S (2017). J. Photochem. Photobiol. B.

[15] Leila Sadeghi FT, Babadi VY (2016). Regul. Toxicol. Pharmacol..

[16] Prasad AS (2016). Mater. Sci. Semicond. Process..

[17] Venkateswarlu S, Natesh Kumar B, Prasad CH, Venkateswarlu P, Jyothi NVV (2014). Phys. B.

[18] Ramanujam K, Sundrarajan M (2014). J. Photochem. Photobiol. B.

[19] Soleymani M, Edrissi M, Alizadeh AM (2015). Polym. J..

[20] Ahn KJ, Lee Y, Kim MS, Im K, Noh S, Yoon H (2015). Sci. Rep..

[21] Lu Y, Yin Y, Mayers BT, Xia Y (2002). Nano Lett..

[22] Mosmann T (1983). J. Immunol. Methods.

[23] Prakash NU, Bhuvaneswari S, Nandhini RS, Azeez NA, Al-Arfaj AA, Munusamy MA (2015). Asian J. Chem..

[24] Matinise N, Fuku XG, Kaviyarasu K, Mayedwa N, Maaza M (2017). Appl. Surf. Sci..

[25] Zeković Z, Kaplan M, Pavlić B, Olgun EO, Vladić J, Canlı O, Vidović S (2016). Ind. Crops Prod..

[26] Sathishkumar P, Preethi J, Vijayan R, Mohd Yusoff AR, Ameen F, Suresh S, Balagurunathan R, Palvannan T (2016). J. Photochem. Photobiol. B.

[27] Mariselvam R, Ranjitsingh AJA, Nanthini AUR, Kalirajan K, Padmalatha C, Selvakumar PM (2014). Spectrochim. Acta A Mol. Biomol. Spectrosc..

[28] Araújo CST, Melo EI, Alves VN, Coelho NMM (2010). J. Braz. Chem. Soc..

[29] Njoku DI, Oguzie EE, Li Y (2017). J. Mol. Liq..

[30] Tronc E, Chaneac C, Jolivet JP (1998). J. Solid State Chem..

[31] Vannerberg NG (1959). Ark. Kemi.

[32] Wang JA, Novaro O, Bokhimi X, López T, Gómez R, Navarrete J, Llanos ME, López-Salinas E (1998). Mater. Lett..

[33] Sayed FN, Polshettiwar V (2015). Sci. Rep..

[34] Rajiv P, Bavadharani B, Kumar MN, Vanathi P (2017). Biocatal. Agric. Biotechnol..

[35] Shahwan T, Abu Sirriah S, Nairat M, Boyaci E, Eroĝlu AE, Scott TB, Hallam KR (2011). Chem. Eng. J..

[36] Smuleac V, Varma R, Sikdar S, Bhattacharyya D (2011). J. Membr. Sci..

[37] Demirci S, Öztürk B, Yildirim S, Bakal F, Erol M, Sancakoğlu O, Yigit R, Celik E, Batar T (2015). Mater. Sci. Semicond. Process..

[38] Wang M, Liu X, Hu J, Li J, Huang J (2015). J. Biomater. Nanobiotechnol..

[39] Bombin S, LeFebvre M, Sherwood J, Xu Y, Bao Y, Ramonell K (2015). Int. J. Mol. Sci..

[40] Ma X, Geisler-Lee J, Deng Y, Kolmakov A (2010). Sci. Total Environ..

[41] Kavya DR, Azeez NA, Deepa VS (2016). Int. J. Res. Pharm. Pharmacother..

[42] Rajendran K, Sen S, Suja G, Senthil SL, Kumar TV (2017). Coll. Surf. B Biointerfaces.

[43] Mahmoud A, Ezgi O, Merve A, Ozhan G (2016). Int. J. Toxicol..

